# Crystal structures of 2-benzyl­amino-4-(4-bromo­phen­yl)-6,7,8,9-tetra­hydro-5*H*-cyclo­hepta­[*b*]pyridine-3-carbo­nitrile and 2-benzyl­amino-4-(4-chloro­phen­yl)-6,7,8,9-tetra­hydro-5*H*-cyclo­hepta[*b*]pyridine-3-carbo­nitrile

**DOI:** 10.1107/S2056989014025936

**Published:** 2015-01-01

**Authors:** R. A. Nagalakshmi, J. Suresh, S. Maharani, R. Ranjith Kumar, P. L. Nilantha Lakshman

**Affiliations:** a Department of Physics, The Madura College, Madurai 625 011, India; b Department of Organic Chemistry, School of Chemistry, Madurai Kamaraj University, Madurai 625 021, India; c Department of Food Science and Technology, University of Ruhuna, Mapalana, Kamburupitiya 81100, Sri Lanka

**Keywords:** crystal structure, cyclo­hepta­[*b*]pyridine, carbo­nitrile, hydrogen bonding, π–π inter­actions

## Abstract

In the title compounds, C_24_H_22_BrN_3_, (I), and C_24_H_22_ClN_3_, (II), the 2-amino­pyridine ring is fused with a cyclo­heptane ring, which adopts a half-chair conformation. The planes of the phenyl and benzene rings are inclined to that of the central pyridine ring [r.m.s. deviations = 0.0083 (1) and 0.0093 (1) Å for (I) and (II), respectively] by 62.47 (17) and 72.51 (14)°, respectively, in (I), and by 71.44 (9) and 54.90 (8)°, respectively, in (II). The planes of the aromatic rings are inclined to one another by 53.82 (17)° in (I) and by 58.04 (9)° in (II). In the crystals of both (I) and (II), pairs of N—H⋯N_nitrile_ hydrogen bonds link the mol­ecules, forming inversion dimers with *R*_2_^2^(12) ring motifs. In (I), the resulting dimers are connected through C—H⋯Br hydrogen bonds, forming sheets parallel to (10-1), and π–π inter­actions [inter-centroid distance = 3.7821 (16) Å] involving inversion-related pyridine rings, forming a three-dimensional network. In (II), the resulting dimers are connected through π–π inter­actions [inter-centroid distance = 3.771 (2) Å] involving inversion-related pyridine rings, forming a two-dimensional network lying parallel to (001).

## Abstract 2

In two cyclo­hepta­[*b*]pyridine-3-carbo­nitrile derivatives, the cyclo­heptane ring adopts a half-chair conformation. In the crystals of both compounds, pairs of N—H⋯N_nitrile_ hydrogen bonds link the mol­ecules, forming inversion dimers with 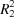
(12) ring motifs.

## Chemical context   

The heterocyclic skeleton containing a nitro­gen atom is the basis of many essential pharmaceuticals and of many physiologically active natural products. Mol­ecules containing heterocyclic substructures continue to be attractive targets for synthesis since they often exhibit diverse and important biological properties. Pyridine is used in the pharmaceutical industry as a raw material for various drugs, vitamins and fungicides, and as a solvent (Shinkai *et al.*, 2000 ▸; Jansen *et al.*, 2001 ▸; Amr *et al.*, 2006 ▸). Pyridines are also omnipresent in medicaments and in agrochemicals (Tomlin, 1994 ▸). Pyridine derivatives have occupied a unique position in medicinal chemistry. Among them, 2-amino-3-cyano­pyridines have been identified as IKK-β inhibitors (Murata *et al.*, 2003 ▸). Many fused cyano­pyridines have also been shown to have a wide spectrum of biological activity (Boschelli *et al.*, 2004 ▸). Our inter­est in the preparation of pharmacologically active 3-cyano­pyridine compounds led us to synthesize the title compounds and we report herein on their crystal structures.
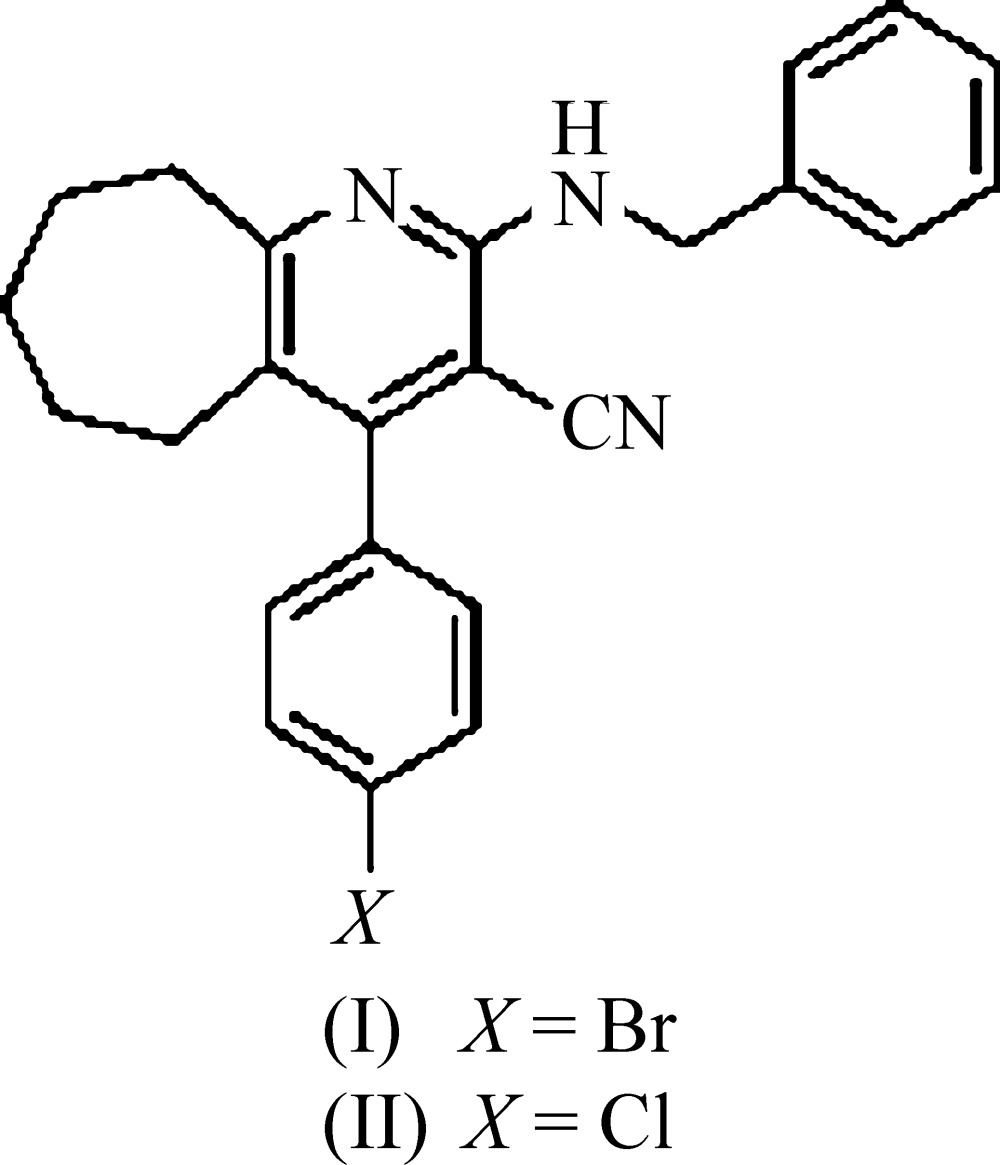


## Structural commentary   

**Figure 1:**
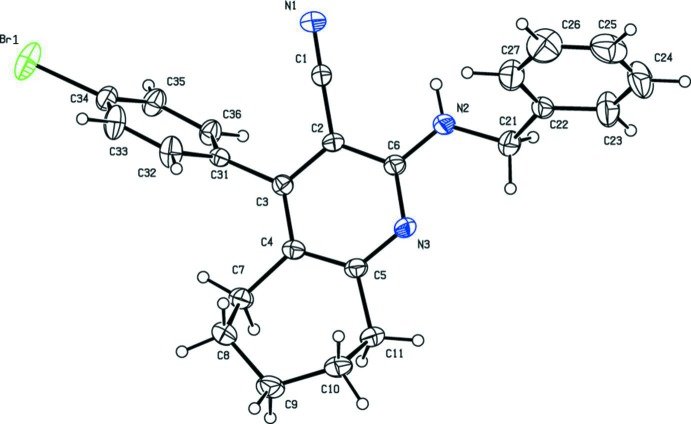
The mol­ecular structure of compound (I), showing 50% probability displacement ellipsoids and the atom labelling.

The mol­ecular structures of the title compounds, (I) and (II), are shown in Figs. 1 ▸ and 2 ▸, respectively. The bromo derivative (I), crystallizes in the monoclinic space group *P*2_1_/*n* while the chloro derivative (II), crystallizes in the triclinic space group *P* 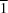.

In both compounds, the pyridine ring is connected to a benzene ring by a –CH_2_—NH_2_– chain, as found in a similar structure *N* ^6^-(4-fluoro­benz­yl)-3-nitro­pyridine-2,6-di­amine (Ge & Qian, 2011 ▸). As expected, the pyridine ring (C2–C6/N3) is planar with r.m.s. deviations of 0.0083 and 0.0093 Å in compounds (I) and (II), respectively. In both compounds, the cyclo­heptane ring adopts a half-chair conformation, with puckering parameters *Q*2 = 0.415 (3) Å, ϕ2 = 310.1 (4)° and *Q*3 = 0.637 (3) Å and ϕ3 = 283.4 (3)° for compound (I) and *Q*2 = 0.475 (2) Å, ϕ2 = 310.3 (2)° and *Q*3 = 0.635 (2) Å and ϕ3 = 283.58 (17)° for compound (II). The amine N atom, N2, attached to the pyridine ring (N3/C2–C6) deviates by only 0.0107 (1) and 0.0073 (1) Å from the ring plane in (I) and (II), respectively. Steric hindrance rotates the benzene ring (C31–C36) out of the plane of the central pyridine ring by 72.51 (14)° in compound (I) and by only 54.90 (8)° in compound (II). The benzene ring is inclined to the phenyl ring (C22–C27) by 53.82 (17) in (I) and by 58.04 (9)° in (II).

## Supra­molecular features   

**Figure 3:**
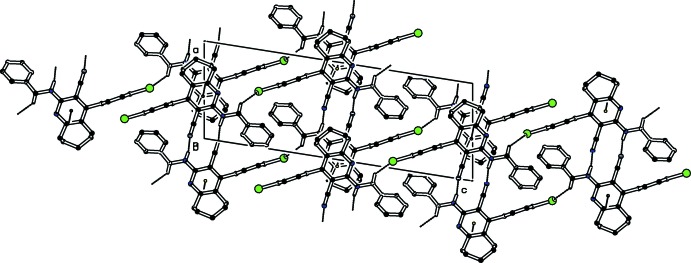
Crystal packing diagram of compound (I), viewed along the *b* axis. Hydrogen bonds (see Table 1 ▸ for details) and π–π inter­actions are shown as dashed lines (centroids are shown as small circles). H atoms not involved in hydrogen bonding have been omitted for clarity.

In the crystal of (I), mol­ecules are linked by pairs of N—H⋯N_nitrile_ hydrogen bonds, forming inversion dimers with 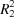
(12) ring motifs (Table 1 ▸ and Fig. 3 ▸). The resulting dimers are connected through C—H⋯Br hydrogen bonds, forming sheets lying parallel to (10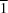). The sheets are connected by weak π–π stacking inter­actions involving adjacent inversion-related pyridine rings with a centroid-to-centroid distance of 3.7710 (7) Å, as shown in Fig. 3 ▸. These inter­actions lead to the formation of a three-dimensional network.

**Figure 4:**
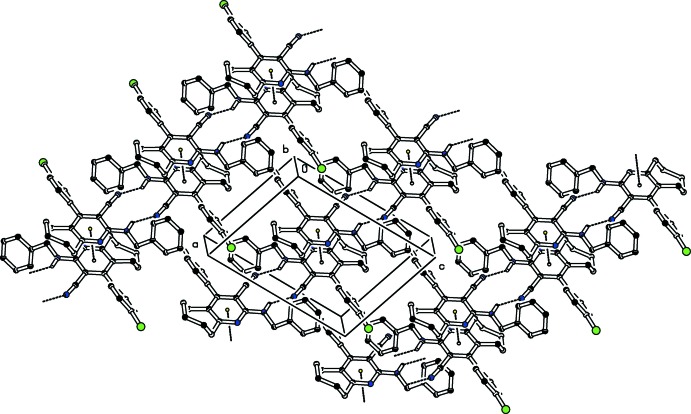
Crystal packing diagram of compound (II), viewed along the *b* axis. Hydrogen bonds (see Table 2 ▸ for details) and π–π inter­actions are shown as dashed lines (centroids are shown as small circles). H atoms not involved in hydrogen bonding have been omitted for clarity.

In the crystal of (II), mol­ecules are also linked by pairs of N—H⋯N_nitrile_ hydrogen bonds, forming inversion dimers with 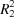
(12) ring motifs (Table 2 ▸ and Fig. 4 ▸). The dimers are connected through weak π–π inter­actions involving inversion-related pyridine rings with a centroid-to-centroid distance of 3.7818 (2) Å (Fig. 4 ▸). The resulting structure is a two-dimensional network lying parallel to (001).

## Synthesis and crystallization   

Compounds (I) and (II) were prepared in a similar manner using 4-bromo aldehyde (1 mmol) for compound (I) and 4-chloro aldehyde (1 mmol) for compound (II). A mixture of cyclo­hepta­none (1 mmol), aromatic aldehyde (1 mmol), malono­nitrile (1 mmol) and benzyl­amine (1mmol) were taken in ethanol (10 ml) to which *p*-TSA (*p*-toluene­sulfonic acid) (1.0 mmol) was added. The reaction mixture was heated under reflux for 2–3 h. On completion of the reaction, verified by thin-layer chromatography (TLC), the mixture was poured into crushed ice and extracted with ethyl acetate. The excess solvent was removed under vacuum and the residue was subjected to column chromatography using a petroleum ether/ethyl acetate mixture (97:3 *v*/*v*) as eluent to afford the pure products. They were recrystallized from ethyl acetate, giving colourless crystals of compounds (I) [m.p. 417 K; yield 74%] and (II) [m.p. 397 K; yield 75%].

## Database survey   

A similar structure reported in the literature, 2-(4-bromophen­yl)-4-(4-meth­oxy­phen­yl)-6,7,8,9-tetra­hydro-5*H*-cyclohepta­[*b*]pyridine (Çelik *et al.*, 2013 ▸) also has a chair conformation of the cyclo­heptane ring and a planar conformation of the pyridine ring, as found for (I) and (II). In compounds (I) and (II) the C—N bond lengths in the –CH_2_—NH_2_– chain, *viz.* C6—N2 and C21—N2, are 1.350 (3) and 1.441 (3) Å, respectively, in (I) and 1.354 (2) and 1.442 (2) Å, respectively, in (II). These distances are similar to those reported for *N* ^6^-(4-fluoro­benz­yl)-3-nitro­pyridine-2,6-di­amine (Ge & Qian, 2011 ▸), *viz*. 1.341 (3) and 1.454 (3) Å, respectively.

## Refinement   

Crystal data, data collection and structure refinement details are summarized in Table 3 ▸. The NH and C-bound H atoms were placed in calculated positions and allowed to ride on their carrier atoms: N—H = 0.86 Å and C—H = 0.93–0.97 Å with *U*_iso_(H) = 1.5*U*_eq_(C) for methyl H atoms and = 1.2*U*_eq_(N,C) for other H atoms.

## Supplementary Material

Crystal structure: contains datablock(s) global, I, II. DOI: 10.1107/S2056989014025936/su5027sup1.cif

Structure factors: contains datablock(s) I. DOI: 10.1107/S2056989014025936/su5027Isup2.hkl

Structure factors: contains datablock(s) II. DOI: 10.1107/S2056989014025936/su5027IIsup3.hkl

Supporting information file. DOI: 10.1107/S2056989014025936/su5027Isup4.cml

Supporting information file. DOI: 10.1107/S2056989014025936/su5027IIsup5.cml

CCDC references: 1036150, 1036149

Additional supporting information:  crystallographic information; 3D view; checkCIF report

## Figures and Tables

**Figure 2:**
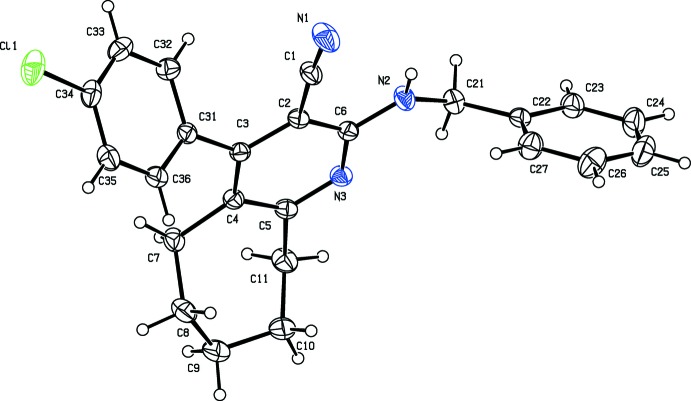
The mol­ecular structure of (II), showing 50% probability displacement ellipsoids and the atom labelling.

**Table 1:** Hydrogen-bond geometry (, ) for (I)

*D*H*A*	*D*H	H*A*	*D* *A*	*D*H*A*
N2H2N1^i^	0.86	2.28	3.010(3)	143
C21H21*B*Br1^ii^	0.97	2.90	3.703(3)	141

Symmetry codes: (i) 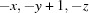; (ii) 
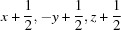
.

**Table 2:** Hydrogen-bond geometry (, ) for (II)

*D*H*A*	*D*H	H*A*	*D* *A*	*D*H*A*
N2H2N1^i^	0.86	2.26	3.007(2)	145

Symmetry code: (i) 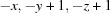.

**Table 3:** Experimental details

	(I)	(II)
Crystal data		
Chemical formula	C_24_H_22_BrN_3_	C_24_H_22_ClN_3_
*M*_r_	432.35	387.89
Crystal system, space group	Monoclinic, *P*2_1_/*n*	Triclinic, *P* 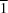
Temperature (K)	293	293
*a*, *b*, *c* ()	8.9710(3), 9.3794(4), 24.9788(9)	9.002(5), 10.097(5), 11.856(5)
, , ()	90, 99.002(2), 90	94.939(5), 108.204(5), 101.272(5)
*V* (^3^)	2075.89(14)	991.3(8)
*Z*	4	2
Radiation type	Mo *K*	Mo *K*
(mm^1^)	1.99	0.21
Crystal size (mm)	0.21 0.19 0.18	0.21 0.19 0.18

Data collection		
Diffractometer	Bruker Kappa APEXII	Bruker Kappa APEXII
Absorption correction	Multi-scan (*SADABS*; Bruker, 2004 ▸)	Multi-scan (*SADABS*; Bruker, 2004 ▸)
*T*_min_, *T*_max_	0.967, 0.974	0.967, 0.974
No. of measured, independent and observed [*I* > 2(*I*)] reflections	51599, 3863, 2927	24808, 3685, 2918
*R*_int_	0.040	0.026
(sin /)_max_ (^1^)	0.606	0.606

Refinement		
*R*[*F* ^2^ > 2(*F* ^2^)], *wR*(*F* ^2^), *S*	0.041, 0.099, 1.10	0.037, 0.105, 1.05
No. of reflections	3863	3685
No. of parameters	253	253
No. of restraints	0	1
H-atom treatment	H-atom parameters constrained	H-atom parameters constrained
_max_, _min_ (e ^3^)	0.42, 0.58	0.19, 0.33

Computer programs: *APEX2* and *SAINT* (Bruker, 2004 ▸), *SHELXS97*, *SHELXL97* and *SHELXL2014* (Sheldrick, 2008 ▸) and *PLATON* (Spek, 2009 ▸).

## supplementary crystallographic information

### Crystal data

**Table:**

C_24_H_22_ClN_3_	*Z* = 2
*M**_r_* = 387.89	*F*(000) = 408
Triclinic, *P*1	*D*_x_ = 1.299 Mg m^−^^3^
*a* = 9.002 (5) Å	Mo *K*α radiation, λ = 0.71073 Å
*b* = 10.097 (5) Å	Cell parameters from 2000 reflections
*c* = 11.856 (5) Å	θ = 2–31°
α = 94.939 (5)°	µ = 0.21 mm^−^^1^
β = 108.204 (5)°	*T* = 293 K
γ = 101.272 (5)°	Block, colourless
*V* = 991.3 (8) Å^3^	0.21 × 0.19 × 0.18 mm

### Data collection

**Table:**

Bruker Kappa APEXII diffractometer	2918 reflections with *I* > 2σ(*I*)
Radiation source: fine-focus sealed tube	*R*_int_ = 0.026
ω and φ scans	θ_max_ = 25.5°, θ_min_ = 2.1°
Absorption correction: multi-scan (*SADABS*; Bruker, 2004)	*h* = −10→10
*T*_min_ = 0.967, *T*_max_ = 0.974	*k* = −12→12
24808 measured reflections	*l* = −14→14
3685 independent reflections	

### Refinement

**Table:**

Refinement on *F*^2^	1 restraint
Least-squares matrix: full	Hydrogen site location: inferred from neighbouring sites
*R*[*F*^2^ > 2σ(*F*^2^)] = 0.037	H-atom parameters constrained
*wR*(*F*^2^) = 0.105	*w* = 1/[σ^2^(*F*_o_^2^) + (0.0486*P*)^2^ + 0.3383*P*] where *P* = (*F*_o_^2^ + 2*F*_c_^2^)/3
*S* = 1.05	(Δ/σ)_max_ < 0.001
3685 reflections	Δρ_max_ = 0.19 e Å^−^^3^
253 parameters	Δρ_min_ = −0.33 e Å^−^^3^

### Special details

**Table:**

Geometry. All e.s.d.'s (except the e.s.d. in the dihedral angle between two l.s. planes) are estimated using the full covariance matrix. The cell e.s.d.'s are taken into account individually in the estimation of e.s.d.'s in distances, angles and torsion angles; correlations between e.s.d.'s in cell parameters are only used when they are defined by crystal symmetry. An approximate (isotropic) treatment of cell e.s.d.'s is used for estimating e.s.d.'s involving l.s. planes.

### Fractional atomic coordinates and isotropic or equivalent isotropic displacement parameters (Å^2^)

**Table:**

	*x*	*y*	*z*	*U*_iso_*/*U*_eq_	
C1	0.1170 (2)	0.47342 (18)	0.36822 (15)	0.0401 (4)	
C2	0.24864 (18)	0.41014 (15)	0.38041 (14)	0.0327 (3)	
C3	0.32569 (17)	0.40885 (15)	0.29430 (13)	0.0304 (3)	
C4	0.44508 (17)	0.33539 (15)	0.30821 (14)	0.0321 (3)	
C5	0.48147 (17)	0.26963 (15)	0.40915 (14)	0.0331 (3)	
C6	0.29611 (17)	0.34315 (15)	0.48044 (14)	0.0320 (3)	
C7	0.53288 (19)	0.32240 (17)	0.21988 (15)	0.0395 (4)	
H7A	0.6478	0.3489	0.2631	0.047*	
H7B	0.5074	0.3854	0.1633	0.047*	
C8	0.4910 (2)	0.17808 (19)	0.14983 (16)	0.0477 (4)	
H8A	0.3760	0.1411	0.1274	0.057*	
H8B	0.5158	0.1839	0.0762	0.057*	
C9	0.5782 (3)	0.0790 (2)	0.21741 (18)	0.0540 (5)	
H9A	0.5446	−0.0086	0.1652	0.065*	
H9B	0.6926	0.1124	0.2336	0.065*	
C10	0.5522 (2)	0.05584 (19)	0.33498 (18)	0.0516 (5)	
H10A	0.6113	−0.0104	0.3685	0.062*	
H10B	0.4389	0.0163	0.3184	0.062*	
C11	0.6037 (2)	0.18367 (19)	0.42923 (16)	0.0451 (4)	
H11A	0.6223	0.1565	0.5081	0.054*	
H11B	0.7046	0.2386	0.4285	0.054*	
C21	0.2814 (2)	0.29636 (18)	0.67846 (15)	0.0416 (4)	
H21A	0.3240	0.3740	0.7428	0.050*	
H21B	0.3692	0.2540	0.6781	0.050*	
C22	0.15556 (19)	0.19495 (16)	0.70552 (14)	0.0347 (3)	
C23	0.1853 (2)	0.16979 (18)	0.82224 (16)	0.0449 (4)	
H23	0.2789	0.2189	0.8823	0.054*	
C24	0.0778 (3)	0.0727 (2)	0.85090 (19)	0.0589 (5)	
H24	0.0996	0.0564	0.9298	0.071*	
C25	−0.0605 (3)	0.0004 (2)	0.7635 (2)	0.0629 (6)	
H25	−0.1323	−0.0658	0.7826	0.075*	
C26	−0.0929 (2)	0.0256 (2)	0.6480 (2)	0.0617 (5)	
H26	−0.1877	−0.0228	0.5886	0.074*	
C27	0.0142 (2)	0.12280 (19)	0.61870 (17)	0.0503 (4)	
H27	−0.0092	0.1396	0.5399	0.060*	
C31	0.28089 (17)	0.49110 (15)	0.19592 (14)	0.0321 (3)	
C32	0.2876 (2)	0.62890 (17)	0.22518 (15)	0.0396 (4)	
H32	0.3223	0.6683	0.3056	0.047*	
C33	0.2437 (2)	0.70820 (18)	0.13681 (17)	0.0459 (4)	
H33	0.2491	0.8004	0.1574	0.055*	
C34	0.19207 (19)	0.64947 (18)	0.01821 (16)	0.0428 (4)	
C35	0.1840 (2)	0.51340 (19)	−0.01437 (16)	0.0454 (4)	
H35	0.1493	0.4748	−0.0950	0.055*	
C36	0.2284 (2)	0.43515 (17)	0.07516 (15)	0.0395 (4)	
H36	0.2231	0.3430	0.0540	0.047*	
N1	0.0094 (2)	0.51850 (19)	0.36511 (16)	0.0616 (5)	
N2	0.22527 (17)	0.34558 (15)	0.56572 (13)	0.0425 (3)	
H2	0.1413	0.3786	0.5517	0.051*	
N3	0.41191 (15)	0.27423 (13)	0.49346 (11)	0.0349 (3)	
Cl1	0.13087 (7)	0.74901 (6)	−0.09170 (5)	0.06667 (19)	

### Atomic displacement parameters (Å^2^)

**Table:**

	*U*^11^	*U*^22^	*U*^33^	*U*^12^	*U*^13^	*U*^23^
C1	0.0457 (9)	0.0481 (10)	0.0398 (9)	0.0242 (8)	0.0226 (7)	0.0144 (7)
C2	0.0336 (7)	0.0323 (8)	0.0369 (8)	0.0132 (6)	0.0151 (6)	0.0062 (6)
C3	0.0315 (7)	0.0271 (8)	0.0337 (8)	0.0084 (6)	0.0118 (6)	0.0038 (6)
C4	0.0294 (7)	0.0317 (8)	0.0382 (8)	0.0096 (6)	0.0141 (6)	0.0050 (6)
C5	0.0307 (7)	0.0328 (8)	0.0362 (8)	0.0110 (6)	0.0104 (6)	0.0028 (6)
C6	0.0328 (8)	0.0297 (8)	0.0369 (8)	0.0093 (6)	0.0153 (6)	0.0047 (6)
C7	0.0368 (8)	0.0452 (9)	0.0472 (9)	0.0170 (7)	0.0224 (7)	0.0144 (8)
C8	0.0523 (10)	0.0567 (11)	0.0441 (10)	0.0229 (9)	0.0246 (8)	0.0062 (8)
C9	0.0662 (12)	0.0486 (11)	0.0619 (12)	0.0279 (9)	0.0335 (10)	0.0071 (9)
C10	0.0646 (12)	0.0436 (10)	0.0629 (12)	0.0302 (9)	0.0318 (10)	0.0148 (9)
C11	0.0454 (9)	0.0549 (11)	0.0453 (10)	0.0294 (8)	0.0172 (8)	0.0146 (8)
C21	0.0435 (9)	0.0471 (10)	0.0368 (9)	0.0101 (8)	0.0168 (7)	0.0088 (7)
C22	0.0398 (8)	0.0331 (8)	0.0381 (8)	0.0154 (7)	0.0184 (7)	0.0066 (6)
C23	0.0513 (10)	0.0473 (10)	0.0398 (9)	0.0150 (8)	0.0171 (8)	0.0111 (8)
C24	0.0745 (14)	0.0608 (13)	0.0570 (12)	0.0228 (11)	0.0345 (11)	0.0288 (10)
C25	0.0589 (12)	0.0536 (12)	0.0923 (16)	0.0156 (10)	0.0404 (12)	0.0339 (12)
C26	0.0479 (11)	0.0535 (12)	0.0764 (14)	0.0035 (9)	0.0144 (10)	0.0156 (11)
C27	0.0498 (10)	0.0527 (11)	0.0455 (10)	0.0092 (9)	0.0127 (8)	0.0119 (8)
C31	0.0298 (7)	0.0341 (8)	0.0386 (8)	0.0116 (6)	0.0167 (6)	0.0094 (6)
C32	0.0413 (9)	0.0368 (9)	0.0408 (9)	0.0121 (7)	0.0123 (7)	0.0060 (7)
C33	0.0460 (9)	0.0332 (9)	0.0594 (11)	0.0111 (7)	0.0164 (8)	0.0136 (8)
C34	0.0384 (9)	0.0483 (10)	0.0500 (10)	0.0146 (8)	0.0195 (8)	0.0240 (8)
C35	0.0499 (10)	0.0558 (11)	0.0370 (9)	0.0171 (8)	0.0196 (8)	0.0115 (8)
C36	0.0457 (9)	0.0380 (9)	0.0416 (9)	0.0158 (7)	0.0206 (7)	0.0072 (7)
N1	0.0655 (10)	0.0839 (13)	0.0638 (11)	0.0493 (10)	0.0377 (9)	0.0285 (9)
N2	0.0472 (8)	0.0511 (9)	0.0466 (8)	0.0259 (7)	0.0281 (7)	0.0202 (7)
N3	0.0366 (7)	0.0351 (7)	0.0366 (7)	0.0145 (6)	0.0135 (6)	0.0072 (6)
Cl1	0.0687 (3)	0.0744 (4)	0.0699 (3)	0.0251 (3)	0.0271 (3)	0.0466 (3)

### Geometric parameters (Å, º)

**Table:**

C1—N1	1.140 (2)	C21—C22	1.505 (2)
C1—C2	1.428 (2)	C21—H21A	0.9700
C2—C3	1.403 (2)	C21—H21B	0.9700
C2—C6	1.408 (2)	C22—C27	1.378 (3)
C3—C4	1.398 (2)	C22—C23	1.381 (2)
C3—C31	1.489 (2)	C23—C24	1.380 (3)
C4—C5	1.399 (2)	C23—H23	0.9300
C4—C7	1.508 (2)	C24—C25	1.366 (3)
C5—N3	1.337 (2)	C24—H24	0.9300
C5—C11	1.505 (2)	C25—C26	1.366 (3)
C6—N3	1.340 (2)	C25—H25	0.9300
C6—N2	1.354 (2)	C26—C27	1.382 (3)
C7—C8	1.529 (3)	C26—H26	0.9300
C7—H7A	0.9700	C27—H27	0.9300
C7—H7B	0.9700	C31—C36	1.388 (2)
C8—C9	1.520 (3)	C31—C32	1.389 (2)
C8—H8A	0.9700	C32—C33	1.380 (2)
C8—H8B	0.9700	C32—H32	0.9300
C9—C10	1.513 (3)	C33—C34	1.373 (3)
C9—H9A	0.9700	C33—H33	0.9300
C9—H9B	0.9700	C34—C35	1.376 (3)
C10—C11	1.524 (3)	C34—Cl1	1.7334 (17)
C10—H10A	0.9700	C35—C36	1.383 (2)
C10—H10B	0.9700	C35—H35	0.9300
C11—H11A	0.9700	C36—H36	0.9300
C11—H11B	0.9700	N2—H2	0.8600
C21—N2	1.442 (2)		
			
N1—C1—C2	174.73 (18)	N2—C21—C22	114.79 (14)
C3—C2—C6	120.15 (13)	N2—C21—H21A	108.6
C3—C2—C1	122.07 (14)	C22—C21—H21A	108.6
C6—C2—C1	117.73 (14)	N2—C21—H21B	108.6
C4—C3—C2	118.39 (14)	C22—C21—H21B	108.6
C4—C3—C31	123.49 (13)	H21A—C21—H21B	107.5
C2—C3—C31	118.06 (13)	C27—C22—C23	118.37 (16)
C3—C4—C5	117.26 (13)	C27—C22—C21	123.14 (15)
C3—C4—C7	123.47 (14)	C23—C22—C21	118.46 (15)
C5—C4—C7	119.26 (13)	C24—C23—C22	120.88 (18)
N3—C5—C4	124.52 (14)	C24—C23—H23	119.6
N3—C5—C11	114.38 (14)	C22—C23—H23	119.6
C4—C5—C11	121.08 (14)	C25—C24—C23	120.05 (19)
N3—C6—N2	118.13 (14)	C25—C24—H24	120.0
N3—C6—C2	120.89 (13)	C23—C24—H24	120.0
N2—C6—C2	120.98 (13)	C24—C25—C26	119.80 (19)
C4—C7—C8	113.51 (14)	C24—C25—H25	120.1
C4—C7—H7A	108.9	C26—C25—H25	120.1
C8—C7—H7A	108.9	C25—C26—C27	120.40 (19)
C4—C7—H7B	108.9	C25—C26—H26	119.8
C8—C7—H7B	108.9	C27—C26—H26	119.8
H7A—C7—H7B	107.7	C22—C27—C26	120.48 (18)
C9—C8—C7	114.77 (15)	C22—C27—H27	119.8
C9—C8—H8A	108.6	C26—C27—H27	119.8
C7—C8—H8A	108.6	C36—C31—C32	118.14 (14)
C9—C8—H8B	108.6	C36—C31—C3	122.71 (14)
C7—C8—H8B	108.6	C32—C31—C3	119.13 (14)
H8A—C8—H8B	107.6	C33—C32—C31	121.05 (16)
C10—C9—C8	116.06 (15)	C33—C32—H32	119.5
C10—C9—H9A	108.3	C31—C32—H32	119.5
C8—C9—H9A	108.3	C34—C33—C32	119.28 (16)
C10—C9—H9B	108.3	C34—C33—H33	120.4
C8—C9—H9B	108.3	C32—C33—H33	120.4
H9A—C9—H9B	107.4	C33—C34—C35	121.39 (16)
C9—C10—C11	115.01 (16)	C33—C34—Cl1	118.72 (14)
C9—C10—H10A	108.5	C35—C34—Cl1	119.86 (14)
C11—C10—H10A	108.5	C34—C35—C36	118.70 (16)
C9—C10—H10B	108.5	C34—C35—H35	120.6
C11—C10—H10B	108.5	C36—C35—H35	120.6
H10A—C10—H10B	107.5	C35—C36—C31	121.43 (16)
C5—C11—C10	113.24 (15)	C35—C36—H36	119.3
C5—C11—H11A	108.9	C31—C36—H36	119.3
C10—C11—H11A	108.9	C6—N2—C21	124.26 (14)
C5—C11—H11B	108.9	C6—N2—H2	117.9
C10—C11—H11B	108.9	C21—N2—H2	117.9
H11A—C11—H11B	107.7	C5—N3—C6	118.73 (13)

### Hydrogen-bond geometry (Å, º)

**Table:**

*D*—H···*A*	*D*—H	H···*A*	*D*···*A*	*D*—H···*A*
N2—H2···N1^i^	0.86	2.26	3.007 (2)	145

Symmetry code: (i) −*x*, −*y*+1, −*z*+1.
